# Integrating functional data analysis with case-based reasoning for hypertension prognosis and diagnosis based on real-world electronic health records

**DOI:** 10.1186/s12911-022-01894-7

**Published:** 2022-06-06

**Authors:** Ping Qi, Fucheng Wang, Yong Huang, Xiaoling Yang

**Affiliations:** 1 Department of Mathematics and Computer Science, Tongling University, Tongling, 244061 China; 2 School of Public Health, Anhui Medical University, Hefei, 230032 China; 3 Tianqiao Community Health Service Station, Tongling Municipal Hospital, Tongling, 244061 China

**Keywords:** Case-based reasoning, Functional data analysis, Time series, Hypertension

## Abstract

**Background:**

Hypertension is the fifth chronic disease causing death worldwide. The early prognosis and diagnosis are critical in the hypertension care process. Inspired by human philosophy, CBR is an empirical knowledge reasoning method for early detection and intervention of hypertension by only reusing electronic health records. However, the traditional similarity calculation method often ignores the internal characteristics and potential information of medical examination data.

**Methods:**

In this paper, we first calculate the weights of input attributes by a random forest algorithm. Then, the risk value of hypertension from each medical examination can be evaluated according to the input data and the attribute weights. By fitting the risk values into a risk curve of hypertension, we calculate the similarity between different community residents, and obtain the most similar case according to the similarity. Finally, the diagnosis and treatment protocol of the new case can be given.

**Results:**

The experiment data comes from the medical examination of Tianqiao Community (Tongling City, Anhui Province, China) from 2012 to 2021. It contains 4143 community residents and 43,676 medical examination records. We first discuss the effect of the influence factor and the decay factor on similarity calculation. Then we evaluate the performance of the proposed FDA-CBR algorithm against the GRA-CBR algorithm and the CS-CBR algorithm. The experimental results demonstrate that the proposed algorithm is highly efficient and accurate.

**Conclusions:**

The experiment results show that the proposed FDA-CBR algorithm can effectively describe the variation tendency of the risk value and always find the most similar case. The accuracy of FDA-CBR algorithm is higher than GRA-CBR algorithm and CS-CBR algorithm, increasing by 9.94 and 16.41%, respectively.

## Introduction

According to the China Cardiovascular Disease Report, there are currently 270 million adult hypertensive patients and 290 million cardiovascular disease patients in China [1]. A systematic analysis of data reveals that China is one of the top nine countries with the most severe rises in both male and female morbidity rates of hypertension [2]. Hypertension has posed a severe threat to public health, and it creates a lot of chain problems. On the one side, hypertensive patients are more likely to develop diabetes, heart failure, angina pectoris, myocardial infarction, and other adverse health outcomes. On the other side, hypertension costs the country about 366 billion in 2020, and the annual cost is rising substantially [3]. However, due to the developing symptoms of hypertension being mostly hidden, most people do not know they have pre-hypertension or already have hypertension. Therefore, the early prognosis and diagnosis is a critical step in the hypertension care process.

Using of data from electronic health record (EHR) has shown great promise for the early detection of chronic disease [4–6]. Various methods have been proposed to provide disease prediction and clinical decision-making aid based on retrospective electronic health records data, such as regression model [7], decision-making tree [8], recurrent neural network [9], and case-based reasoning (CBR) [10]. Inspired by human philosophy, CBR is an empirical knowledge reasoning method to find the recorded case most relevant to the target case, which avoids the training process of machine learning algorithms. The recorded case that has occurred in the past is referred to as the source case, and the source cases are used to guide the solution of the target case. Because of its better learning capability and interpretability than the rule-based and model-based reasoning algorithm, CBR has been widely used in medical diagnosis [11].

**Fig. 1:**
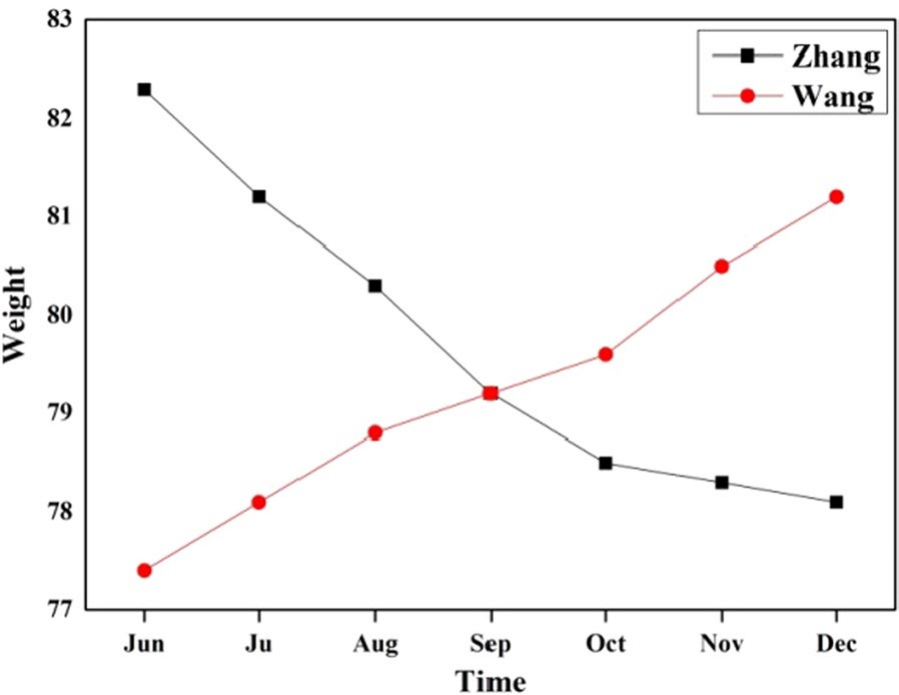
Weight changes of Zhang and Wang during half a year

As mentioned above, the core idea of CBR is to handle similar problems. In the medical field, the diagnosis and treatment protocol of the new case can be given according to the most similar source case in the case base. However, the traditional CBR algorithms tend to focus on cross-sectional study in the medical field and ignore the impact of time. As shown in Fig. 1, the weights of Zhang and Wang are the same in September. But we do not consider that the importance of A and B remain the same in October. The main reason is that the various tendencies of two people are significantly different. Obviously, the similarity between source case and target case is also affected by time. The more recent the history information is, the more impact the time factor has.

In addition, although the community health service stations organize free medical examinations every half year, some community residents do not attend continuously. Therefore, for real-world electronic health records, the time and frequency of medical examination for different people may be different as well. In this case, how to evaluate the similarity between two people is of great importance.

Because of the aforementioned limitations mentioned above, we first analyze and evaluate the risk value of hypertension based on the medical examination data of community resident. As each community resident has multiple medical examination records, these data are fitted into a risk curve of hypertension for each resident based on functional data analysis (FDA) technique. Then, we can calculate the similarity between two different curves, which can be defined as the similarity between the recorded case and the target case. Finally, a novel FDA-based CBR model named FDA-CBR is proposed in this paper.

The remainder of this paper is listed as follows: “Related work” section presents the related work. “Material and method” section proposes the material and method. “Discussion” section demonstrates the experimental results. Finally, the conclusions and improvement directions are presented in section “Conclusions”.

## Related work

Since Roger Schank first came up with CBR in 1982, CBR has become one of the prominent reasoning mechanisms, and has been widely used in medical diagnosis, industrial production and engineering planning. Using the previous experience or knowledge, CBR can realize the reuse of essential knowledge and effectively extract the complex rules. The processing of the CBR system contains case reuse, case retrieve, case revise, and case retain.

As is well-known, the similarity calculation between the target case and the source cases is the critical step for case retrieval. Therefore, similarity calculation has become a research hotspot recently. Euclidean distance function and Mahalanobis distance function are the most commonly used distance functions of similarity in many studies [12, 13].

Some studies integrate Cosine similarity or Jaccard similarity into the CBR system, which makes the applicability of similarity measure of the traditional CBR widely extended. Zhang et al. [14] calculate the angle between two eigenvectors by using the cosine theorem and Euclidean distance, which is defined as the Cosine similarity. In the meantime, the traditional single-category attribute is extended to relative entropy model-based multi-attribute. Baharav et al. [15] use Jaccard distance to measure similarity between sample sets. Min-hashes are employed to efficiently estimate these similarities. Chen et al. [16] present an emergency decision model based on grey relational analysis, which can effectively quantify the attribute weights and the similarity for the heterogeneous multi-attributes decision-making problem.

In addition, there are so many risk factors for hypertension, i.e. obesity, smoking, alcohol consumption and waistline. A reasonable weight assignment of attributes has a significance influence on the decision result. Multivariate logistic regression model [17] and cox regression model [18] are the most representative risk prediction model. However, with the growth of data in volume and dimensionality, the ability of data mining algorithms to deal with mass-data becomes more important. Some classification based data mining technique, such as random forest [19] and SVM [20], has performed well for multilabel classification using knowledge-driven features. It also can reduce the complexity of the model by reducing the number of features required to train a machine learning model.

In recent years, with the rapid development of machine learning techniques, some machine learning algorithms have been used to learn the similarity between two record cases. Zhang et al*.* [21] adopt the earth mover’s distance as the similarity between two dense images, which is used for classification. Vij et al*.* [22] present a machine learning-based approach to find out the similarity between two texts. Unfortunately, although machine learning technique-based algorithms are very useful, these algorithms are not widely used due to lack of samples.

In summary, distance measure function based similarity calculation method can only reflect the relationship in spatial location, but ignores the time series and variation tendency of the record cases. Fortunately, functional data analysis is a statistical analysis technique especially suited for the analysis of curves, which can be used for s table estimates and accurate predictions [23]. To overcome the above shortcomings, functional data analysis is a suitable method to capture the time series similarity of two data series in the system.

## Material and method

This study received ethical approval from the Ethics Committee of Tongling Municipal Hospital and Anhui Medical University. The study was performed in compliance with the World Medical Association Declaration of Helsinki on Ethical Principles for Medical Research Involving Human Subjects, and research regulations of the country. Considering retrospective nature of the study, Informed consent was waived by the Ethics Committee of Tongling Municipal Hospital.

### Material

The data comes from the medical examination of the Tianqiao Community (Tongling City, Anhui Province, China) from 2012 to 2021. It contains 4143 community residents and 43,676 medical examination records. Each record includes more than 100 attributes, such as demographic information, physical examination, physiology, biochemistry, and so on. As shown in Table 1, the quantization assignment method is One-Hot encoding. Because of the community health service station organizes free medical examination in March and September each year, the time of medical examination can be marked with its ordinal number. For example, the medical examination in March 2012 is marked as “1”. Similarly, September 2012 and September 2021 can be marked as “2” and “20” separately.

**Table 1:** Medical examination information

Variable	Quantitative assignment
Hypertension	NO→1; YES→1
Gender	Female→0;Male→1
Age	> 65→1; 35 ~ 65→2; < 35→3
Exercise frequency	Never→1; Everyday→2; Once a week or more→3; Occasionally→4
Dietary habit	Meat diet→1; Vegetarian diet→2; Equilibrium→3
Smoking	Yes→1; Never→2; Quitting→3
Drinking	Everyday→1; Frequently→2; Never→3; Occasionally→4
Heart rhythm	Normal→0; Arrhythmia→1
Central obesity	< 90 cm(Male) or < 80 cm(Female)→0; > 90 cm(Male) or > 80 cm(Female)→1
BMI	18.5 ~ 24→1; 24 ~ 28→2; > 28→3; < 18.5→4
Diabetes	No→0; Yes→1
Heart rate	60 ~ 100→1; > 100→2; < 60→3
Blood urea	3.2 ~ 7.1→1; > 7.2→2; < 3.2→3
**…**	**…**
Total cholesterol	> 5.2→1; 3.0 ~ 5.2→2; < 3.0→3
Triglyceride	< 1.7→1; 1.7 ~ 5.65→2; ≥ 5.65→3
Low-density lipoprotein	< 4.14→0; ≥ 4.14→1
High-density lipoprotein	≥ 1.2→0; < 1.2→1

### Method

In this subsection, we describe the novel FDA-based CBR model in detail. Firstly, we calculate the weights of input attributes by random forest algorithm. Secondly, for every community resident, the risk value of hypertension from each medical examination can be evaluated according to the input data and the attribute weights. Then, these continuous or not completely continuous risk values are fitted into a curve of risk value by using the medical examination time as the variable. Based on this, we can calculate the similarity between two curves, and the most similar case is extracted according to the similarity. Finally, the diagnosis and treatment protocol of the new case can be given.

**Fig. 2:**
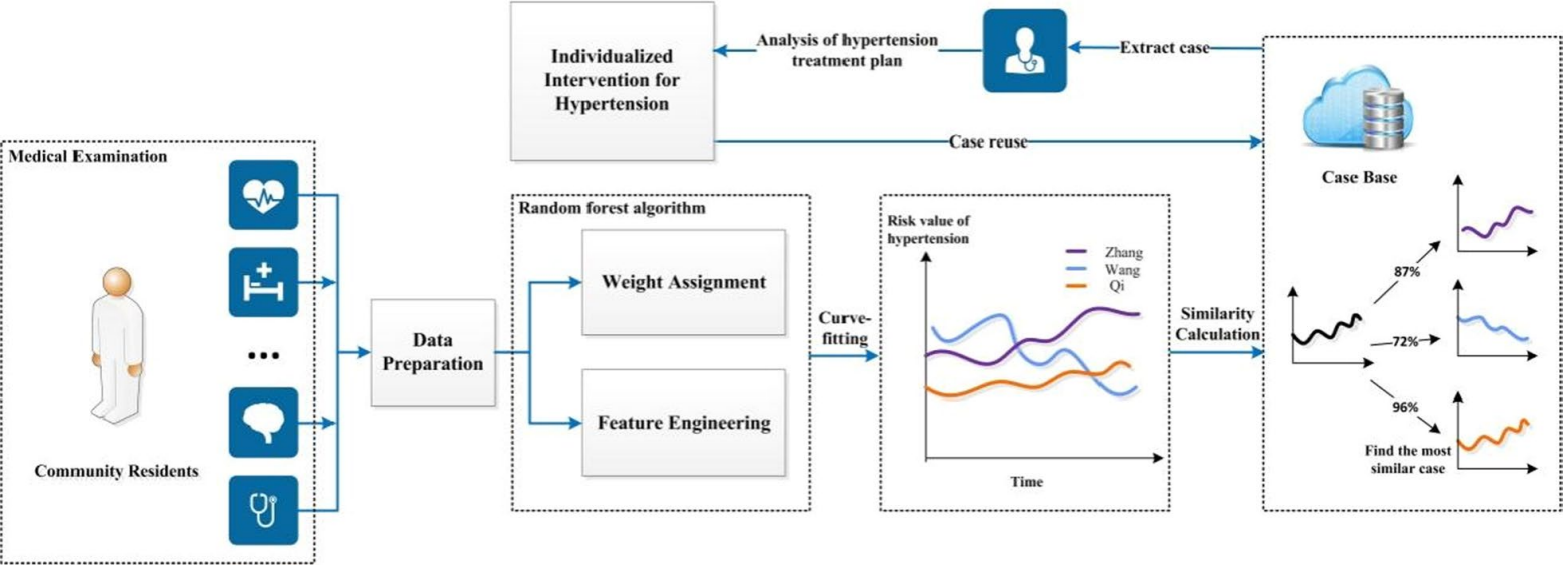
Whole workflow of FDA-CBR

The case extract strategy is as follows: when the similarity between the new case and the source case is over 90%, we can directly reuse the diagnosis and treatment protocol of the source case. When the similarity is between 70 and 90%, the diagnosis and treatment protocol of the source case can be regarded as an alternative treatment plan. When the similarity is between 60 and 70%, the extracted case can be used as an auxiliary reference plan. The whole workflow of FDA-CBR is shown in Fig. 2.

#### Weight assignment based on random forest algorithm

**Fig. 3:**
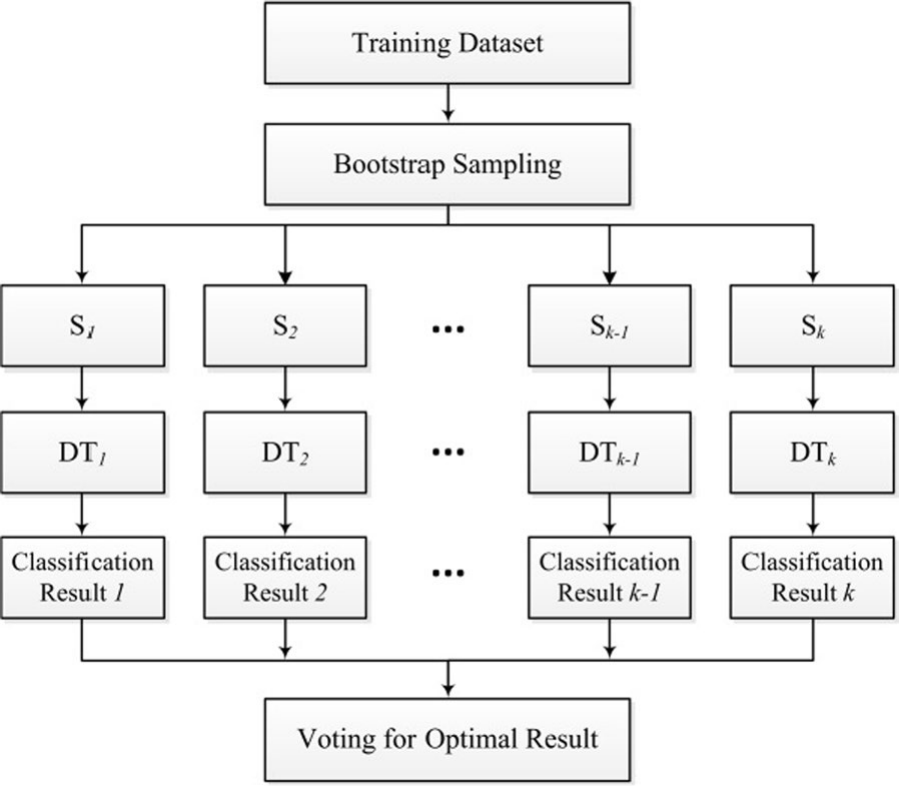
Workflow of random forest algorithm

In order to make the weight calculation of attributes more reasonable, a random forest algorithm is employed in this paper. Random forest algorithm combines different decision trees (decision tree, DT). Each decision tree depends on the values of independently sampled random vectors. As shown in Fig. 3, the weight assignment is obtained by casting a vote for the most effective class. Assuming that each case is represented by an *n*-dimensional feature vector *X* = {*x*_*1*_,*x*_*2*_,…,*x*_*n*_}, the weight vector of the attributes can be described as follows: *W* = {*w*_*1*_, *w*_*2*_,…, *w*_*n*_}. The algorithm flow is listed as follows:

1. *S* = {*S*_*1*_,*S*_*2*_,…,*S*_*k*_} are sampled randomly from the medical examination data, and the bootstrap sampling method is employed in this process;
2. Different decision trees are constructed based on *S*. During the construction phase of each decision tree, when the value of *Gini*(*t*) increases, less available information can be gained. Therefore, the total *Gini* value of all derived nodes should be less than that of the parent node. The minimum *Gini* value is used as the best splitting standard of the nodes, calculated by Formula (1).
  1 $$Gini\left( t \right) = 1 - \mathop \sum \limits_{j = 1}^{k} \left[ {p\left( {j{|}t} \right)} \right]^{2}$$
  where *p*(*j*|*t*) denotes the probability of risk class *j* at node *t*.
3. Each decision tree votes for the most effective classification, and the vote results determine the optimal weight assignment. Assuming that *D*_*i*_ is the mean *Gini* decrease for *i*-th variable. *w*_*i*_ is an *i*-th variable weight, which can be calculated by Formula (2):
  2 $$w_{i} = \frac{{D_{i} }}{{\mathop \sum \nolimits_{i = 1}^{n} D_{i} }}$$

#### Curve-fitting and similarity calculation

Based on weight assignment, the risk value of hypertension from the *i*-th medical examination can be evaluated according to the input data and the attribute weights by Formula (3).

3 $$risk_{i} = X \times W = \left( {x_{1} ,x_{2} , \ldots x_{n} } \right) \times \left( {w_{1} ,w_{2} , \ldots w_{n} } \right)$$

For community residents, each of them may have multiple medical examination results. Therefore, these dynamically changing risk values are fitted into a curve of risk value by using the medical examination time as the variable. Then, we can calculate the similarity between the two curves. The specific calculation steps are as follows: basis function selection, smoothing function, calibration function, and similarity calculation.

(1) Basis function

Basis function fitting is the most common method of FDA. Basis function is a series of independent function, which is defined as $R\left( t \right) = \mathop \sum \limits_{k = 1}^{K} c_{k} \phi_{k} \left( t \right)$, where *Φ*_*k*_(*t*) (*k *= 1, 2,…, *K*) are *k* selected basis functions, *c*_*k*_ is the coefficient matrix. In general, the B-spline basis function is more appropriate for aperiodic functional data. Assuming that the time interval [1, 20] (medical examination from March 2012 to September 2021) is divided into several subintervals [*t*_*i-1*_,*t*_*i*_], where *t*_*i*_ is the time of *i*-th medical examination, *risk*_i_ is the risk value of hypertension from *i*-th medical examination, 1 ≤ *t*_*0*_ < *t*_*1*_ < … < *t*_*N*_ ≤ 20. *B*_*i,k*_(*t*) is defined recursively as the B-spline basis function of order *k* by Formula (4) and Formula (5).

4 $$B_{{i,0}} \left( t \right) = \left\{ {\begin{array}{*{20}c} {1,} & {t_{i} \le t \le t_{{i + 1}} } \\ {0,} & {{\text{otherwise}}} \\ \end{array} } \right.$$

5 $$B_{i,k} \left( t \right) = \frac{{t - t_{i} }}{{t_{i + k} - t_{i} }}B_{i,k + 1} \left( t \right) + \frac{{t_{i + k + 1} - t}}{{t_{i + k + 1} - t_{i + 1} }}B_{i + 1,k - 1} \left( t \right)$$

(2) Smoothing function

According to the basis function, the coefficient vector should be calculated by the least square method. That is to minimize the following formula.

6 $${\text{SMSSE}}\left( {Risk|C} \right) = \mathop \sum \limits_{j = 1}^{n} \left[ {risk_{j} - \mathop \sum \limits_{k = 1}^{K} c_{k} \phi_{k} \left( {t_{j} } \right)} \right]^{2}$$

where *Risk* and *C* are the matrix form of {*risk*_*j*_}, {*c*_*k*_}.

(3) Calibration function

Unlike point data, the properties of functional data include amplitude and phase. Therefore, the purpose of the calibration function is to move the misaligned variable to the same standard by adjusting the translation parameters. Let the translation parameter be *δ*_*i*_, *R*_i_^*^(*t*) = *R*_*i*_(*t* + *δ*_*i*_), *δ*_*i*_ can be calculated by minimizing Formula (7).

7 $$REGSSE = \mathop \sum \limits_{i = 1}^{n} \mathop \smallint \limits_{{t_{1} }}^{{t_{2} }} \left[ {R_{i} \left( {t + \delta_{i} } \right) - \hat{\mu }\left( t \right)} \right]^{2} dt = \mathop \sum \limits_{i = 1}^{n} \mathop \smallint \limits_{{t_{1} }}^{{t_{2} }} \left[ {R_{i}^{*} \left( {t + \delta_{i} } \right) - \hat{\mu }\left( t \right)} \right]^{2} dt$$

where $\hat{\mu }\left( t \right)$ is the mean value of all the functional data in [*t*_*1*_,*t*_*2*_]. This mean value function is updated iteratively until it stabilizes, which makes the translation parameter more rational.

(4) Similarity calculation

The resampling technique is used in this paper to collect data. Firstly, we transform the risk scores into functional data using FDA, and then define the continuity of data exactly by using function properties. In the calculation process, the fitting function is divided into 19 intervals: {(1,2),(2,3),…(19,20)}. We can obtain the continuous function in any one of these intervals. Then the interval similarities are calculated separately and integrated into the global similarity with decay factor, which makes the calculation more accurate.

The interval similarity between two curves is calculated in two parts: actual distance and derived function distance. The actual distance describes the data discrepancy, and the derived function distance describes the discrepancy of inherent characteristics. Let *R*_*org*_(*t*) and *R*_*tgt*_(*t*) be the functional descriptions of the original case and target case, respectively. Then the actual distance *d*_*act*_ between *R*_*org*_(*t*) and *R*_*tgt*_(*t*) on [*t*_*1*_,*t*_*2*_] can be calculated by Formula(8).

8 $$d_{{act}} = \sqrt[2]{{\int_{{t_{1} }}^{{t_{2} }} {\left( {R_{{org}} \left( t \right) - R_{{tgt}} \left( t \right)} \right)^{2} dt} }}$$

Let *R′*_*org*_(*t*) and *R′*_*tgt*_(*t*) be the derived functions of *R*_*org*_(*t*) and *R*_*tgt*_(*t*) respectively. Then the derived function distance *d*_*der*_(*R′*_*org*_(*t*), *R′*_*tgt*_(*t*)) on [*t*_*1*_,*t*_*2*_] can be calculated by Formula(9)

9 $$d_{{der}} = \sqrt[2]{{\int_{{t_{1} }}^{{t_{2} }} {\left( {R_{{org}}^{\prime } \left( t \right) - R_{{tgt}}^{\prime } \left( t \right)} \right)^{2} dt} }}$$

Thus, two kinds of distance between the original case and target case can be aggregated into an integrated similarity *Sim*(*org*, *tgt*) as follows:

10 $$Sim\left( {org,tgt} \right) = \theta \cdot d_{act} + \left( {1 - \theta } \right) \cdot d_{der} , \;\;\theta \in (0,1)$$

where *θ* is the influence factor of actual distance and derived function distance. Briefly, *θ* ∈ (0, 0.5) indicates that we are more concerned with the variation tendency of the risk value of medical examination data.

(5) Decay factor

As discussed above, recent information has more influence on similarity calculation. The decay factor *μ* is employed to reflect the importance of the historical information, which decreases as time pass on. The similarity with decay factor can be calculated as follows:

11 $$Sim\left( n \right) = \sum\nolimits_{{i = 1}}^{n} {Sim_{i} \times \mu ^{{\left( {n - i} \right)}} }$$

## Discussion

### Weight assignment

With the aid of the grid searching technique (GridSearchCV), the depth and number of decision trees are set to 5 and 500 separately. The experimental result is shown in Table 2.

**Table 2:** Weights of input attributes

Variable	Weights of input attributes
Age	0.301
Diabetes	0.152
Exercise frequency	0.112
BMI	0.103
Total cholesterol	0.073
Smoking	0.051
Drinking	0.049
Central obesity	0.041
Triglyceride	0.032
Blood urea	0.031
Serum high lipoprotein cholesterol	0.028
Heart rhythm	0.027
Gender	0.024
Heart rate	0.023
Serum low lipoprotein cholesterol	0.019
Dietary habit	0.017
**…**	**…**

As shown in Table 2, the top 10 weighted attributes are age, diabetes, exercise frequency, BMI, total cholesterol, smoking, drinking, central obesity, triglyceride, and blood urea. According to Formula (3), the risk value of hypertension from each medical examination can be evaluated according to the input data and the attribute weights. Table 3 shows a community resident’s risk value of hypertension in 10 consecutive medical examinations.

**Table 3:** Risk value of hypertension in 10 continuous medical examinations

Time	2017.3	2017.9	2018.3	2018.9	2019.3	2019.9	2020.3	2020.9	2021.3	2021.9
Risk value	13.6	13.2	13.8	14.5	15.6	15.9	15.4	15.8	15.7	16.2

In the real world, this community resident is 52 years old. He does not drink or smoke. However, he seldom does exercise, and his BMI has been increasing since 2017. He was diagnosed with hypertension in 2019. The experimental result shows that the risk value can effectively reflect the hypertension risk of community residents.

### Influence factor

**Fig. 4:**
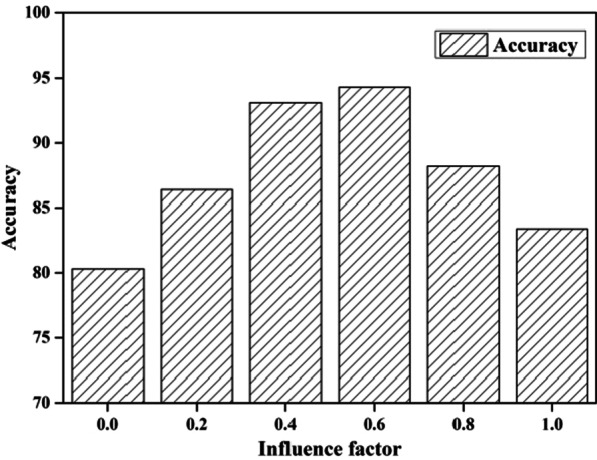
Effect of influence factor

In this experiment, we discuss the effect of the influence factor *θ* in Formula (8). In order to evaluate the performance of the proposed algorithm, we define “correct result” as follows: The doctors select 10 cases from the case base that are most similar to the target case in their mind. When the calculation result is one of these five cases, the calculation result is marked as “correct”. If not, it’s marked as “incorrect”. On this basis, we randomly select 100 community residents, and then we can obtain the accuracy of CBR-based algorithms by comparing the calculation results and the doctors. In this experiment, the decay factor is set to 0.8. Figure 4 shows the experiment results.

As shown in Fig. 4, when *θ* = 0.6, the accuracy is the highest of various situations. As the value of the influence factor increases or decreases, the accuracies are significantly decline. When *θ* = 0, the actual distance between curves is not considered, the accuracy is lowest. When *θ* = 1, the derived function distance has no effect on the similarity calculation. The accuracy is just a little higher than *θ* = 0. The experiment results indicate that both actual distance and derived function distance have a significant impact on similarity calculation. *θ* is set to 0.6 in the following experiments.

### Decay factor

**Fig. 5:**
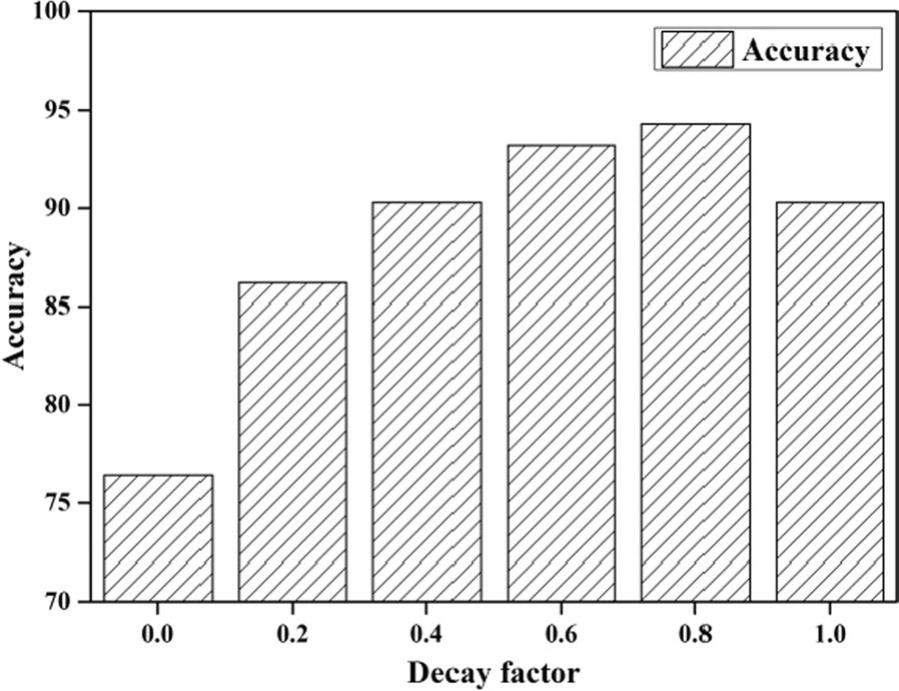
Effect of decay factor

In this experiment, we consider the effect of decay factor *μ* on similarity calculation. Figure 5 shows the experiment results.

As shown in Fig. 5, when *μ* = 0, the accuracy is the lowest of various situations. The main reason is that the similarity calculation method ignores the history records and the variation tendency. Only the latest history record can be considered. As the decay factor increases, the accuracy increases as well. The experiment results illustrate that the delay factor can effectively reflect the influence of time on similarity calculation. In the meantime, when *μ* is greater than 0.8, the accuracy is a little lower. This experiment results indicate that the recent records have more impact than the old records, and the old records gradually lose their reference value. *μ* is set to 0.8 in the following experiment.

### Performance evaluation of proposed algorithm

**Fig. 6:**
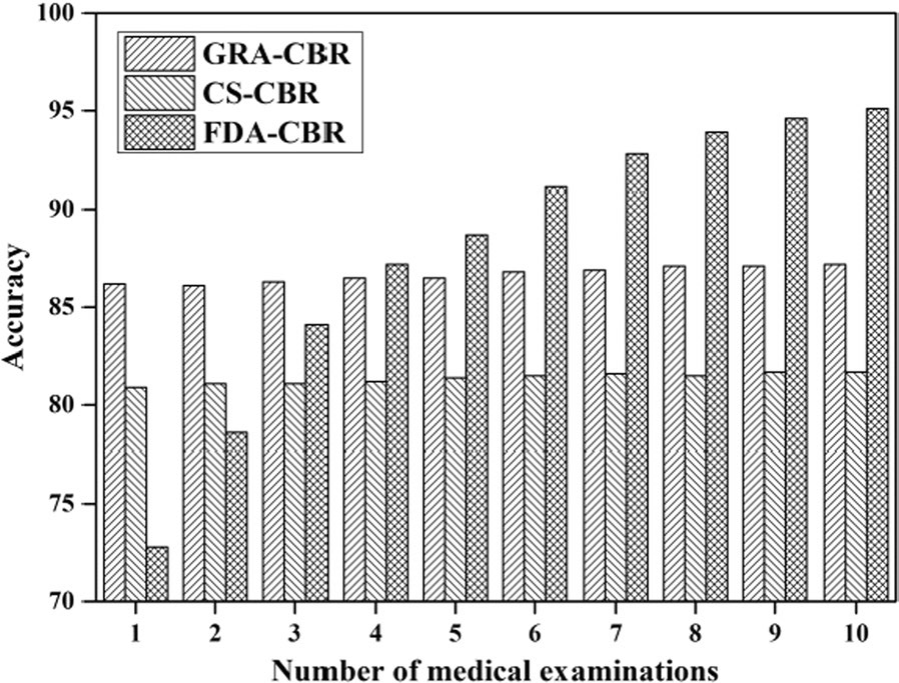
Comparison of accuracy of FDA-CBR, CS-CBR, and GRA-CBR under varying number of medical examinations

In this experiment, we evaluate the performance of the proposed FDA-CBR algorithm against the GRA-CBR algorithm [16] and CS-CBR algorithm [14]. GRA-CBR algorithm is a grey relational analysis (GRA) based similarity calculation algorithm, which enables the CBR to quantify the similarity with heterogeneous multi-attributes and makes the attribute weights assignment more reasonable by considering information correlation. CS-CBR algorithm is a decision model based on Cosine similarity and Euclidean distance. Figure 6 shows the experiment results.

In Fig. 6, with the number of medical examinations of the new case increases, the accuracy of FDA-CBR increases as well. However, GRA-CBR and CS-CBR are designed without consideration of the variation tendency of the risk value. Therefore, the change in the number of medical examinations has little impact on the accuracy of GRA-CBR and CS-CBR.

In the meantime, it’s worth mentioning that the accuracy of proposed algorithm is lower than two other algorithms when the number is less than or equal 2. However, when the number is greater than 4, the accuracy of FDA-CBR is significantly higher than GRA-CBR and CS-CBR. The accuracy of FDA-CBR is 9.94 and 16.41% higher than GRA-CBR and CS-CBR when the number is equal to 10. The main reason is that it is hard to identify the most similar cases just by only one or two recent medical examination. The variation tendency of the risk value is difficult to describe when there is no enough data. At this time, reducing the value of influence factor is beneficial to improve the accuracy. In addition, the experiment results indicate that the proposed FDA-CBR algorithm can effectively reveal the internal characteristics of the medical examination data and find the most similar case. It provides an effective method for the establishment of personalized intervention model for hypertension and other chronic diseases.

## Conclusions

Hypertension has posed a severe threat to public health, and it creates a lot of chain problems. In this paper, we present a novel FDA-based CBR model. Firstly, the weights of input attributes are calculated by random forest algorithm. Then, the risk value of hypertension from each medical examination is evaluated according to the input data and the attribute weights. By fitting the risk values into a risk curve of hypertension, we calculate the similarity between two curves and obtain the most similar case according to the similarity. The experiment results show that the accuracy of FDA-CBR algorithm is higher than GRA-CBR and CS-CBR, increasing by 9.94% and 16.41% respectively. It provides an effective method for the establishment of personalized intervention model for hypertension and other chronic diseases.

However, as mentioned above, when the number of medical examinations of the new case is less than 3, the accuracy of FDA-CBR is a little lower than GRA-CBR. Therefore, how to adjust the similarity calculation process with the lack of input data is our future work.

## Data Availability

The data that support the findings of this study are available from Tongling Municipal Hospital but restrictions apply to the availability of these data, which were under license for the current study. Data are available from the corresponding author upon reasonable request and with permission of the Ethics Committee of Tongling Municipal Hospital and the Ethics Committee of Anhui Medical University.

## Abbreviations

HER: Electronic health record
CBR: Case-based reasoning
FDA: Functional data analysis
DT: Decision tree
GRA: Grey relational analysis
